# Training bacteria to produce environmentally friendly polymers of industrial and medical relevance

**DOI:** 10.1111/1751-7915.13470

**Published:** 2019-08-05

**Authors:** María José Huertas, Miguel A. Matilla

**Affiliations:** ^1^ Instituto de Bioquímica Vegetal y Fotosíntesis Universidad de Sevilla‐CSIC Américo Vespucio 49 41092 Sevilla Spain; ^2^ Department of Environmental Protection Estación Experimental del Zaidín Consejo Superior de Investigaciones Científicas Prof. Albareda 1 Granada 18008 Spain

The high demand of the increasing world population is resulting in the fast depletion of natural resources as well as challenging the long‐term sustainability of natural ecosystems. This is clearly reflected in the environmental impact associated with the use of petroleum‐based plastic materials. For instance, 8.3 billion tonnes of synthetic plastics were globally produced since the early 1950s (Geyer *et al*., 2017) and current estimates indicate that their production will reach 33 billion tonnes by 2050 (Rhodes, 2018). While not even 10% of the global plastic waste is annually recycled (Brooks *et al*., 2018), the remaining plastic waste ends up in landfills, oceans or is incinerated (Drzyzga and Prieto, 2019), which results in enormous amounts of recalcitrant residues contaminating and accumulating in the environment. Consequently, the development of environmentally safe materials that replace petroleum‐based plastics is urgently needed and biopolymers are among the best alternative to be considered.

Biopolymers are polymeric biomolecules synthesized by animals, plants and microorganisms, but also include those polymers that are chemically produced from renewable resources (i.e. corn, sugar, starch; Niaounakis, 2015). These biopolymers stand out due to their biodegradability, biocompatibility and functional versatility. In fact, among their broad range of applications, biopolymers are currently used for numerous medical, dental and pharmaceutical purposes, cosmetics and electronics, as well as clothing fabrics, food additives and industrial plastics (Freitas *et al*., 2011; Niaounakis, 2015; Schmid *et al*., 2015; Avila Rodriguez *et al*., 2018; García and Prieto, 2019; Jacek *et al*., 2019; Portela *et al*., 2019; Wroblewska‐Krepsztul *et al*., 2019).

Since the first discovery of a bacterial polymer in the nineteenth century (Pasteur, 1861), a vast number of new biopolymers was identified (Rehm, 2010; Freitas *et al*., 2011; Schmid *et al*., 2015). Based on their composition, bacterial polymers are categorized into four main classes: polysaccharides (e.g. xanthan, dextran, alginate, cellulose), polyesters (e.g. polyhydroxyalkanoates), polyanhydrides (e.g. inorganic polyphosphate) and polyamides (e.g. cyanophycin, poly‐γ‐glutamate) (Rehm, 2010; Freitas *et al*., 2011). However, the advancement of genomics and genome mining strategies continues to reveal the extraordinary genetic potential of bacteria as a source of novel biopolymers. Among other benefits, the production of these polymers provides bacteria with protection against biotic and abiotic stresses, as well as serving as storage of carbon and energy (Rehm, 2010; Perez‐Mendoza and Sanjuan, 2016).

Two of the main medically and industrially relevant biopolymers worldwide are cellulose and alginate. The global market for these two polymers currently amounts to over USD 22 billion per year, with projections to reach USD 50 billion by 2025 (Anderson *et al*., 2018). At present, plants and algae are almost exclusively the sole sources of commercially available cellulose and alginate, respectively (Hay *et al*., 2013; Heinze, 2016; García and Prieto, 2019). However, multiple bacteria have been found to produce these polymers and the commercial potential of bacterial cellulose (BC; also known as bacterial nanocellulose) and alginate has extensively been recognized (Hay *et al*., 2013; Portela *et al*., 2019). Thus, for example, BC shows great potential as a novel wound healing promoting material and several BC‐based medical and cosmetic products are commercially available, including CELMAT^®^ from Bowil Biotech (Poland), BASYC^®^ from POLYMET Jena (Germany) and Securian^®^ from Xylos (USA). Nevertheless, the major bottleneck currently restricting industrial production and applicability of bacterial polymers is associated with their high production costs (Mozejko‐Ciesielska and Kiewisz, 2016; Azeredo *et al*., 2019). Therefore, in order to improve the production yield of bacterial polymers, variables such as the bacterial producer strain and its growth conditions must be optimized (Azeredo *et al*., 2019; Jacek *et al*., 2019). Furthermore, the composition of the culture media not only affects production yields but also determines the morphology of the biomaterials and their physical properties (Singhsa *et al*., 2018; Azeredo *et al*., 2019). This is illustrated in the case of BC since there are still many drawbacks to scale up the production of this biopolymer and to reduce its currently high production costs. Importantly, physical properties (i.e. polymerization, fibril diameter, thermal stability) of BC are still unexplored and since they are key determinants influencing the final application of the biopolymer (Singhsa *et al*., 2018; Azeredo *et al*., 2019)*,* research in this area is needed. With this aim, Chen and co‐workers recently evaluated the impact of different carbon sources derived from plant biomass on the productivity and quality of BC produced by the model bacterium *Komagataeibacter xylinus* ATCC 23770. In this report, the authors demonstrated that bacteria grown in culture media based on glucose or maltose achieved higher productivity, polymerization degree and thermal stability as compared with bacteria grown on the same media supplemented with alternative sugars (e.g. xylose, mannose or galactose). Remarkably, when the strain ATCC 23770 was grown in a medium containing a sugar mixture that mimics a lignocellulosic hydrolysate, BC productivity and yield were enhanced but other parameters like fibril diameter, polymerization degree, crystallinity and thermal stability were reduced (Chen *et al*., 2019). Overall, further investigations are required to link sugar metabolism with BC yield and quality – a research field with evident biomedical and industrial applications.

The biosynthesis of polymers is energetically demanding for bacteria, and their production is frequently highly regulated, with many biosynthetic gene clusters being cryptic under standard culture conditions (Rehm, 2010; Hay *et al*., 2014; Schmid *et al*., 2015; Perez‐Mendoza and Sanjuan, 2016). As a result, future synthetic biology strategies will be focused not only on developing *à‐la‐carte* bacterial polymers with specific chemical and physical properties but also on the construction of bacterial strains with improved production yields (Anderson *et al*., 2018). This issue has been addressed by Valentine and co‐workers in a recent report published in Microbial Biotechnology using *Pseudomonas aeruginosa* PAO1 as model bacterium (Valentine *et al*., 2019). The major exopolysaccharide produced by this strain is alginate (Maunders and Welch, 2017). However, *P. aeruginosa* is a common opportunistic pathogen, which limits its use as a source of commercial alginate. In addition, the synthesis of this polymer by *P. aeruginosa* is tightly controlled (Hay *et al*., 2014). One of the key players in this complex regulatory circuit is MucE, an outer membrane protein that when overexpressed leads to an increased expression of the alginate biosynthetic gene cluster (Qiu *et al*., 2007). In order to use *P. aeruginosa* as a potential producer of commercial alginate, Valentine and co‐workers engineered the strain *P. aeruginosa* PGN5, in which five key virulence genes of PAO1 were deleted. These deletions did not affect the capacity of PGN5 to synthesize alginate, either at the structural or at the concentration levels. Indeed, MucE overexpression in PGN5 resulted in overproduction of alginate, even at higher levels than when MucE was overexpressed in the wild‐type strain. Remarkably, the authors showed that PGN5 was avirulent in mice models with no mortality observed after 4 weeks post‐infection. In contrast, 95% and 20% mortality were quantified within 48 h when mice were injected with wild‐type *P. aeruginosa* and the Food and Drug Administration (FDA) approved *Escherichia coli* strain BL21, respectively. By tagging the genomes of PAO1 and PGN5 with the *lux* operon, the authors demonstrated that the avirulent phenotype of PGN5 was associated with its impaired ability to spread in mice models since no bacterial dissemination from the injection site was observed. Altogether, given the reduced toxicity and invasiveness of PGN5, Valentine *et al*. (2019) highlighted the potential use of this strain not only as an alternative to algae as a commercial source of alginate but also as a microbial cell factory for the production of alginates with specific chemical and physical properties.

Despite improvements in metabolic engineering and synthetic biology strategies for microbial synthesis of biopolymers, the development of novel technologies to improve bacterial production yields from natural sources is still a challenge. For example, redirecting carbon fluxes and the use of novel gene‐editing technologies like CRISPR‐Cas9 are potential strategies to be exploited. Exploring novel sequences and protein functions will additionally be a powerful route to generate new functional biomaterials by synthetic biology methods (Anderson *et al*., 2018). However, parameters such as energy and material efficiency and process economics need to be optimized to consider bacterial polymer production as a green technology (Anderson *et al*., 2018). Remarkably, the use of toxic aromatic hydrocarbons (e.g. naphthalene) as a carbon source for biopolymer production has been successfully explored recently (Marin *et al*., 2019). This approach will lay the foundation for coupling the bioremediation of toxic compounds with the generation of value‐added biopolymers exhibiting a wide range of biotechnological applications.

## Conflict of interest

None declared.
